# Negative and positive urgency as pathways in the intergenerational transmission of suicide risk in childhood

**DOI:** 10.3389/fpsyt.2024.1417991

**Published:** 2024-09-23

**Authors:** Ana Ortin-Peralta, Amara Schiffman, Jill Malik, Lillian Polanco-Roman, Laura Hennefield, Katherine Luking

**Affiliations:** ^1^ Ferkauf Graduate School of Psychology, Yeshiva University, Bronx, NY, United States; ^2^ Department of Psychiatry and Behavioral Sciences, Albert Einstein College of Medicine, Bronx, NY, United States; ^3^ Department of Psychology, The New School, New York, NY, United States; ^4^ Department of Psychiatry, Washinton University School of Medicine in St. Louis, St. Louis, MI, United States; ^5^ Department of Psychology, Saint Louis University, St. Louis, MO, United States

**Keywords:** suicidal ideation, suicide attempts, familial transmission, impulsivity, UPPS-P, childhood

## Abstract

**Introduction:**

Parental suicide attempts and suicide death increase suicide risk in their offspring. High levels of impulsivity have been observed in families at high risk for suicide. Impulsivity, a highly heritable trait that is especially elevated in childhood, is frequently measured with the UPPS-P Impulsive Behavior Scale, which includes negative urgency, positive urgency, sensation seeking, premeditation, and perseverance. Our study examined the association between the UPPS-P facets and suicide ideation (without suicide attempts) and suicide attempts at baseline and first-time endorsement within the next two years in childhood. We also examined how the UPPS-P facets mediated the association between parental suicide attempts and suicide death and offspring first-time suicide ideation and attempts at follow-up.

**Methods:**

The sample was 9,194 children (48.4% female; 9-10 years old) from the Adolescent Brain Cognitive Development (ABCD) study, assessed yearly three times. At Time 1 (T1), caregivers reported on suicide attempts and suicide deaths (combined) of the biological parents. Caregivers and children reported on suicide ideation and attempts in the KSADS-PL DSM-5 at each time point, T1 and follow-up (T2 and/or T3). The Short UPPS-P Scale (child-report) assessed the impulsivity facets at T1, which were computed as latent variables.

**Results:**

At T1, 6.7% of children had a parent who had attempted or died by suicide. Most UPPS-P facets were associated with suicide ideation and attempts at T1 and T2/T3. In adjusted models, parental suicide attempts and suicide death were associated with offspring negative and positive urgency. In mediation models, parental suicide attempts and suicide death had an indirect effect on offspring first-time suicide ideation at T2/T3 through negative urgency (OR = 1.04; 95% CI, 1.01-1.08) and positive urgency (OR = 1.03, 95% CI, 1.01-1.05). Similar results were found for first-time suicide attempts at T2/T3.

**Discussion:**

Our findings support an impulsive pathway in the familial transmission of suicide risk. For all youth, interventions that target multiple UPPS-P facets may help prevent or reduce suicide risk. For offspring whose parents have attempted or died by suicide, clinicians should pay particular attention to children who impulsively act on extreme emotions, as they may be at higher suicide risk.

## 1 Introduction

Childhood suicide is on the rise in the US, where suicide is the 5^th^ leading cause of death for children ages 5-12 years since 2017 (1). The age group of 10-to-14-year-olds experienced the largest increase in suicide deaths from 1999 to 2014 compared to other age groups (2). From 2007 to 2015, children ages 5-11 accounted for 42% of emergency department visits for suicide ideation (SI) and suicide attempts (SAs) among 5-to-18-year-olds (3), highlighting the scope of suicide risk in childhood. Thus, it is imperative to identify early precursors of suicide risk to improve the detection and assessment of children at high risk for suicide and inform developmentally appropriate intervention targets.

Having a parent who has ever attempted suicide or died by suicide increases the offspring’s risk for SI and SA (4–6) and is linked to attempting suicide earlier relative to offspring whose parents have never attempted suicide or died by suicide (7–9). The elevated levels of impulsivity observed in clinical samples of individuals with high family loading for suicidal behavior suggest that impulsivity may be a potential pathway in the transmission of suicide risk (8, 10, 11). However, the direct association between parental SA and suicide death (SD) and impulsivity in their children has not been examined. Genetic and environmental factors support this association. Impulsivity is a highly heritable trait, with genetic effects especially pronounced in childhood (12, 13). Furthermore, parental mental health problems are well-known risk factors for child maltreatment (14–16), which in turn are linked to heightened impulsivity in the offspring (17) and subsequent suicide risk (18). A recent study has also documented family conflict as a potential pathway through which parental mental health problems may increase impulsivity in their offspring (19). Additional support comes from studies that have focused on offspring externalizing problems, which are characterized by manifestations of poor impulsive control, such as high levels of impulsivity (20–22). Studies show an association between parental SA and suicide death (SD) and offspring externalizing problems across the lifespan, including alcohol or substance use disorders, ADHD, and delinquent behavior (23–28). All together, these findings suggest that parental SA/SD may be associated with offspring impulsivity, which in turn may play a role in the familial transmission of suicide risk.

Elevated levels of impulsivity have been observed among individuals with suicidal thoughts and behaviors across the lifespan (29, 30), or who have died by suicide, especially at younger ages (31). However, trait impulsivity is a broad and heterogeneous construct that captures different developmental processes with unique behavioral manifestations (32). From Whiteside and Lynam (2001) (33) and subsequent work (34, 35) emerged the UPPS-P model and associated scale to parse out the heterogeneity of trait impulsivity. This model proposed five facets: negative urgency (“the tendency to act rashly in response to distress or negative affect”), positive urgency (“the tendency to act rashly in response to extreme positive affect”), lack of premeditation (“the tendency to act without thinking”), lack of perseverance (“the inability to remain focused on a task”), and sensation seeking (“the tendency to seek out novel and thrilling experiences”) (35, p. 807; 36, p. 3). Adult studies using the UPPS-P model, which are largely cross-sectional and mostly focused on SI, have found significant associations between the impulsivity facets and suicide risk in adjusted models (29). Negative urgency has received the strongest support, showing a significant association with both SI and SA in models that account for other variables (37–40) and at least one additional UPPS-P facet (41–45). While the association between lack of premeditation and lack of perseverance and SI has received some support (37–39, 43, 46, 47), the association between sensation seeking and suicidal thoughts and behaviors is less robust (37–39, 41–43, 48, 49). The few studies that include positive urgency provide initial evidence for its association with SI and SA (39, 49).

Despite the elevated levels of impulsivity in childhood (50), only three studies have examined the UPPS-P model in youth. In two adolescent samples, emotion-based impulsivity, but not lack of premeditation, was significantly associated with SAs (44, 51). The only study with a child sample focused on negative and positive urgency and found that both facets were cross-sectionally associated with lifetime SI (52). As such, no prior studies have examined the cross-sectional and longitudinal associations between the five impulsivity facets and suicide risk in childhood.

Regarding the possible association between parental SA/SD and the UPPS-P facets, the few studies that have examined childhood maltreatment or adverse childhood experiences (ACEs), which include parental death and psychopathology, have identified significant associations between those adversities and perseverance and, especially, negative urgency (17, 53).

**Figure 1:**
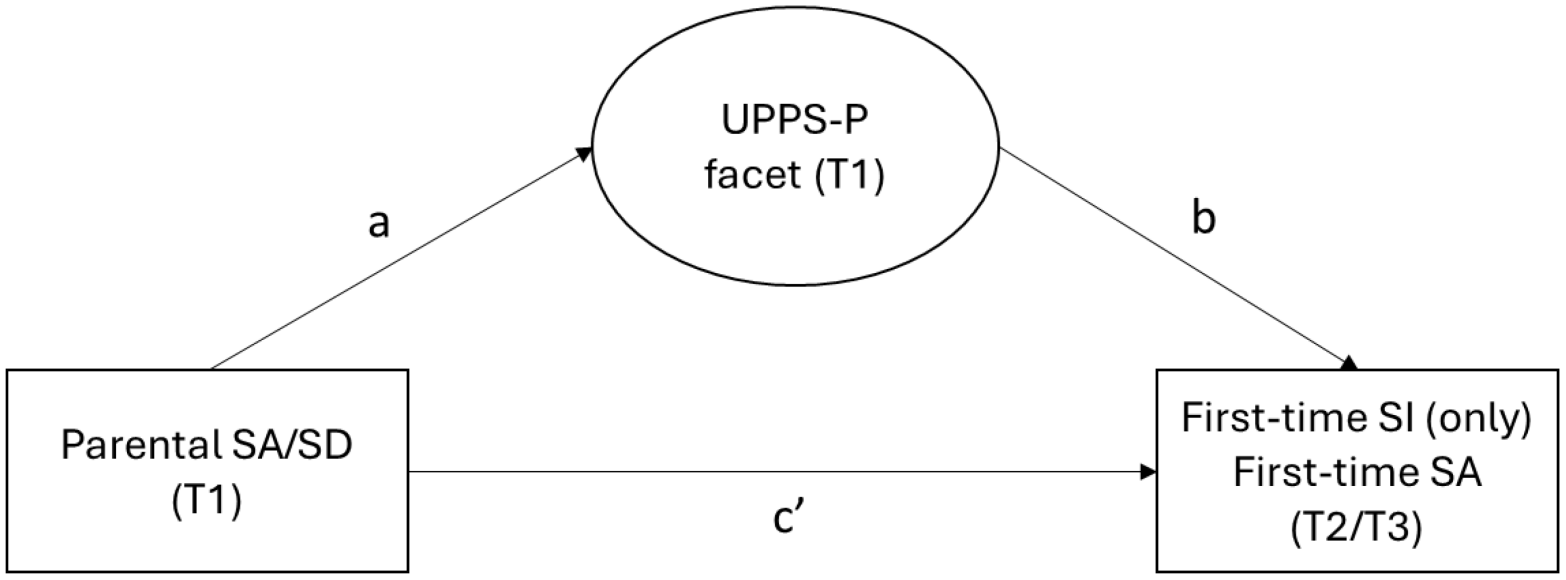
Mediation model testing the a path (from predictor to mediator), the b path (from mediator to outcome, adjusting for predictor) and the c’ path (from predictor to outcome, adjusting for mediator). Offspring first-time suicide ideation excludes children who had ever attempted suicide. SI, suicide ideation; SA, suicide attempt; SD, suicide death; T, time.

Ascertaining the role of the five impulsivity facets on suicidal thoughts and behaviors and the intergenerational transmission of suicide risk is needed to inform developmentally appropriate prevention and intervention responses to break the intergenerational cycle. Thus, this study tested the association between the UPPS-P facets and lifetime SI and SA at baseline, and first-time endorsement of SI and SA during the next two years. A second aim of the study was to examine how the UPPS-P facets mediated the association between parental SA/SD and offspring first-time endorsement of SI and SA at follow-up (**Figure 1**). For all UPPS-P facets, we anticipated finding a cross-sectional and prospective association between negative urgency and offspring SI and SA. This impulsivity facet would mediate the association between parental SA/SD and offspring first-time SI and first-time SA. In the absence of studies in youth and the lack of prospective studies, we did not have specific hypotheses about how the other UPPS-P facets would relate to parental SA/SD and offspring SI and SA.

## 2 Materials and methods

### 2.1 Sample

The study included a subsample of 9,194 children (48.4% female; 55.5% non-Hispanic White) from the Adolescent Brain Cognitive Development (ABCD) study, a US population-based longitudinal study of 9 to 10 years old children (n = 11,868 at baseline or time 1). At time 1 (T1), children were 47.8% female, 52.0% non-Hispanic White, 15.0% non-Hispanic Black, 20.3% Hispanic, 2.1% non-Hispanic Asian, 10.5% non-Hispanic Other Races (unweighted proportions). The ABCD sample was largely recruited through public, private, and charter elementary schools. A population neuroscience approach to recruitment was adopted by employing epidemiologically informed procedures to ensure demographic variation in its sample (54, 55). Eligibility for the ABCD Study was determined by each of the 22 research sites. Details about the study procedures, sampling, and recruitment are described in prior work (54, 56, 57). Caregivers provided informed consent and permission for the child’s participation in the study and children provided assent.

In the current study, we included data from the first three assessments, T1 (baseline), T2 (1-year assessment) and T3 (2-year assessment), conducted between 2016 and 2021. Children with complete data on parental SA/SD at T1 and the suicide outcomes across assessments were included in this study (see **Supplementary Figure 1**). The final sample size was 9,194. The informants were biological mothers (87.4%), biological fathers (9.8%), adoptive parent (0.7%), custodial parent (0.7%) and others (1.3%). Missing values in the covariates ranged between 0 (Race/ethnicity) and 59 (Family structure). Compared to included children, excluded children (n = 2,674) had a higher proportion of non-Hispanic Black, Hispanic, or non-Hispanic other (*p* <.001), single-caregiver family (*p* <.001), and internalizing problems (*p* = .008). Excluded children also experienced more financial adversity (*p* = .002). They did not differ in lifetime SI (with no SA) (*p* = .148), lifetime SA (*p* = .243), parental SA/SD (*p* = .974), and biological sex (*p* = .207) at T1.

The Institutional Review Board (IRB) of Yeshiva University (WCG) approved the study procedures. The ABCD Study was approved by the central IRB of the University of California.

### 2.2 Measures

#### 2.2.1 Parental history of SA/SD

At T1, caregivers reported about the lifetime history of SA or suicide death of each one of the biological parents on the Family History Assessment Module Screener (FHAMS) (58, 59): “Has any blood relative of your child ever attempted or committed suicide?” Positive responses for biological mothers and fathers were combined such that if either parent had attempted suicide or died by suicide, parental SA/SD was coded as ‘yes.’

#### 2.2.2 Offspring suicide ideation and suicide attempts

On a computerized version of the Kiddie Schedule for Affective Disorders and Schizophrenia–Present and Lifetime Version (K-SADS-PL) – DSM-5, children (at T1, T2 and T3) and caregivers (at T1 and T3) reported on the presence or absence of past and current SI and SA at each yearly assessment. These questions were not asked to caregivers at T2. The K-SADS-PL DSM-5 is a structured interview used to assess criteria for psychiatric disorders according to the Diagnostic and Statistical Manual of Mental Disorders Fifth Edition (DSM-5; American Psychiatric Association, 2013) (60, 61). For each informant, the presence of either past or current SI or SA was combined and coded as lifetime SI or lifetime SA, respectively, at each assessment (T1, T2, and T3). The study included four outcomes and two time periods: baseline (T1) and follow-up (T2 and/or T3). At T1, we examined presence of ‘lifetime SA’, and presence of lifetime SI with no lifetime SA, which was coded as ‘lifetime SI (only)’. Regarding the outcomes at follow-up, children with no lifetime SI at T1 who endorsed lifetime SI at T2 and/or T3 and absence of a lifetime SA at any assessment were coded as ‘first-time SI (only)’ at T2/T3. Children with no lifetime SAs at T1 who endorsed a lifetime SA at T2 and/or T3 were coded as ‘first-time SA’ at T2/T3 (62).

#### 2.2.3 Impulsivity facets

At T1, children completed the Short UPPS-P Impulsive Behavior Scale, a self-report scale with 20 items rated on a four-point Likert scale: (1) disagree strongly, (2) disagree some, (3) agree some, and (4) agree strongly (63–65). Items assess the five facets described in the UPPS-P model, negative urgency, positive urgency, (lack of) premeditation (reverse coded), (lack of) perseverance (reverse coded), and sensation seeking. The Short UPPS-P Scale has shown good validity, reliability, and measure invariance in youth (63, 66–68). Exploratory and confirmatory factor analyses with children and adolescent samples support the five-factor structure proposed in the model (63, 65, 68, 69). Twenty-three cases were excluded due to missing data in all or almost all items. Of the included children, ten children were missing one or two items (**Supplementary Figure 1**).

#### 2.2.4 Covariates

We selected socio-demographic and clinical variables that have been associated with impulsivity, SI or SA in childhood, including child’s biological sex, race and ethnicity, family structure, child’s internalizing problems, and financial adversity (50, 52, 62, 70). These variables were reported by caregivers at T1. Race and ethnicity were combined in one unique variable with four categories: Non-Hispanic White, Non-Hispanic Black, Hispanic, Non-Hispanic Other (including Asian, American Indian/Alaska Native, and other). Based on prior research, non-Hispanic Black was used as the reference category (62). Financial adversity was assessed through caregiver report, using a seven-item checklist that assesses the inability to meet basic needs due to financial problems in the past year. The scores on this questionnaire reflect the total number of items endorsed (No/Yes) (e.g., inability to access medical care, lack of sufficient access to food, having gas or electricity shut off for nonpayment, inability to pay rent or mortgage). A total mean score was calculated by summing and averaging the seven items (range: 0-1). If a participant had four or more missing items out of the seven items, their total score was coded as missing (*n* = 17). Caregivers answered a question about their marital/living arrangements. Their responses were dichotomized to capture ‘family structure’ as married/co-habiting *vs*. single-caregiver family (i.e., single, widowed, divorced, separated). Children internalizing problems were assessed via caregiver report using the Child Behavior Checklist for Ages 6-18 years (CBCL/6-18). Items include problems with anxiety, depression, withdrawal, and somatic complaints in the past 6 months. The items, scales, and norms were scored following the ASEBA Manual (71). Raw scores were converted to t scores. A t score of 65 or higher was used as the clinical cutoff point. Using the date of interview at T3 (2-year assessment), we created a dichotomous variable to identify children who completed T3 assessment after the COVID-19 pandemic was officially declared worldwide on March 11, 2020 (72). All variables, except for COVID-19, were associated with at least one of the outcomes and were included as covariates (**Supplementary Table 1**).

### 2.3 Data analysis

First, we described the prevalence of parental SA/SD, outcomes, and covariates. Rao-Scott adjusted chi-squared test was used to examine the association between parental SA/SD and lifetime SI (only) and lifetime SA at T1, and first-time SI (only) and first-time SA at T2/T3. Logistic regression analyses were used to examine the association between parental SA/SD and each outcome, adjusting for child’s sex, race/ethnicity, internalizing problems, family structure, and financial adversity.

Confirmatory factor analysis (CFA) was used to identify shared variation in the UPPS-P items as measured via latent factor scores to reduce measurement error (73). The goodness-of-fit indices used to assess model fit were the root mean square error of approximation (RMSEA, a measure of absolute fit that tests the difference between the model and the data per model degrees of freedom), the Comparative Fit Index (CFI, an indicator of fit compared to the null model), and the SRMR (Standardized Root Mean Square Residual, a measure of the discrepancy between the observed and predicted covariance matrix). Model fit was deemed adequate if the RMSEA was ≤.05, CFI was ≥.95, and SRMR was <.05 (74, 75). When models needed to be adjusted based on these criteria, covariance standardized residuals were examined for values greater than +/- 2.5. Of the two indicators with the largest standardized residual and modification indices, the indicator whose removal improved the goodness-of-fit statistics the most was removed from the model. Given our large sample size, we did not use the values of the χ2 test and associated p and degrees of freedom to assess model fit because its significance is highly sensitive to the size of the sample (76).

The univariate associations between each UPPS-P facet (latent variable) and each outcome, lifetime SI (only) and lifetime SA at T1, and first-time SI (only) and first-time SA at T2/T3, were examined using structural equation modeling (SEM) (Model 1). Each model was further adjusted for child’s sex, race/ethnicity, internalizing problems, family structure, and financial adversity (Model 2). In Model 3, the five facets were entered simultaneously, adjusting for the covariates.

Finally, causal mediation modeling using SEM was used to calculate the indirect effect of parental SA/SD on first-time SI (only) and first-time SA at T2/T3 through each UPPS-P facet (**Figure 1**) (77). In models adjusting for the covariates, we first examined the association between parental SA/SD and each UPPS-P facet (a path) and between the UPPS-P facets and each outcome adjusting further for parental SA/SD (b path). Then, we examined the association between parental SA/SD and first-time SI (only) and first-time SA at T2/T3, adjusting for each UPPS-P facet (c’ path). The indirect effect of parental SA/SD through each UPPS-P facet was tested using bootstrapping with a resampling of 100. Causal mediation with dichotomous outcomes can only be tested with one mediator at a time (77).

Analyses were conducted in MPlus (Version 8.10). We accounted for the survey procedures using ranked propensity scores as weights and research sites as clusters (provided by the ABCD study at T1) in all the analyses. The inclusion of weights allows for the evaluation of less biased estimates to compensate for underrepresentation or overrepresentation within the sample based on individual and household variables (i.e., age, sex, race/ethnicity, family income, marital status, household size, parents’ work force, and Census Region) (55). We used data from the ABCD Annual Release 4.0 and 5.0 (78, 79).

## 3 Results

### 3.1 Parental SA/SD and offspring suicide ideation (only) and suicide attempts

The prevalence of the study variables is displayed in **Table 1**. In this sample, 6.7% percent of children had a biological parent who had attempted suicide or died by suicide at T1: mothers (n = 314, 4.0%), fathers (n = 196, 2.4%) or both (n = 24, 0.3%). At T1, 13% of children reported lifetime SI and 1.2% lifetime SA. At T2/T3 (ages range: 9.7-13.8 years old), 8.9% of children reported first-time SI and 1.7% reported a SA for the first time.

**Table 1:** Prevalence of the study variables.

	Unweighted count	Weighted % (SE)
Outcomes		
Lifetime SI (only) at T1	1,183	13.0 (0.6)
Lifetime SA at T1	93	1.2 (0.1)
First-time SI (only) at T2/3	842	8.9 (0.3)
First-time SA at T2/3	147	1.7 (0.2)
Parental SA/SD		
No	8,660	93.4 (0.7)
Yes	534	6.6 (0.7)
Sex		
Female	4,361	48.4 (0.6)
Male	4,832	51.5 (0.6)
Race/ethnicity		
Non-Hispanic Black	1,179	11.4 (2.3)
Non-Hispanic White	5,095	55.8 (5.8)
Hispanic	1,812	23.1 (6.1)
Non-Hispanic Other	1,108	9.6 (1.4)
Family structure		
Single-caregiver family	2,183	30.4 (2.3)
Married/cohabiting	6,952	69.6 (2.3)
COVID-19		
No	6,689	73.8 (1.7)
Yes	2,498	26.2 (1.7)
Internalizing problems		
No	8,391	90.3 (0.8)
Yes	801	9.7 (0.8)
Financial adversity		
M (SD)	0.07	0.01

SI, suicide ideation; SA, suicide attempt; SD, suicide death; T, time.

**Figure 2:**
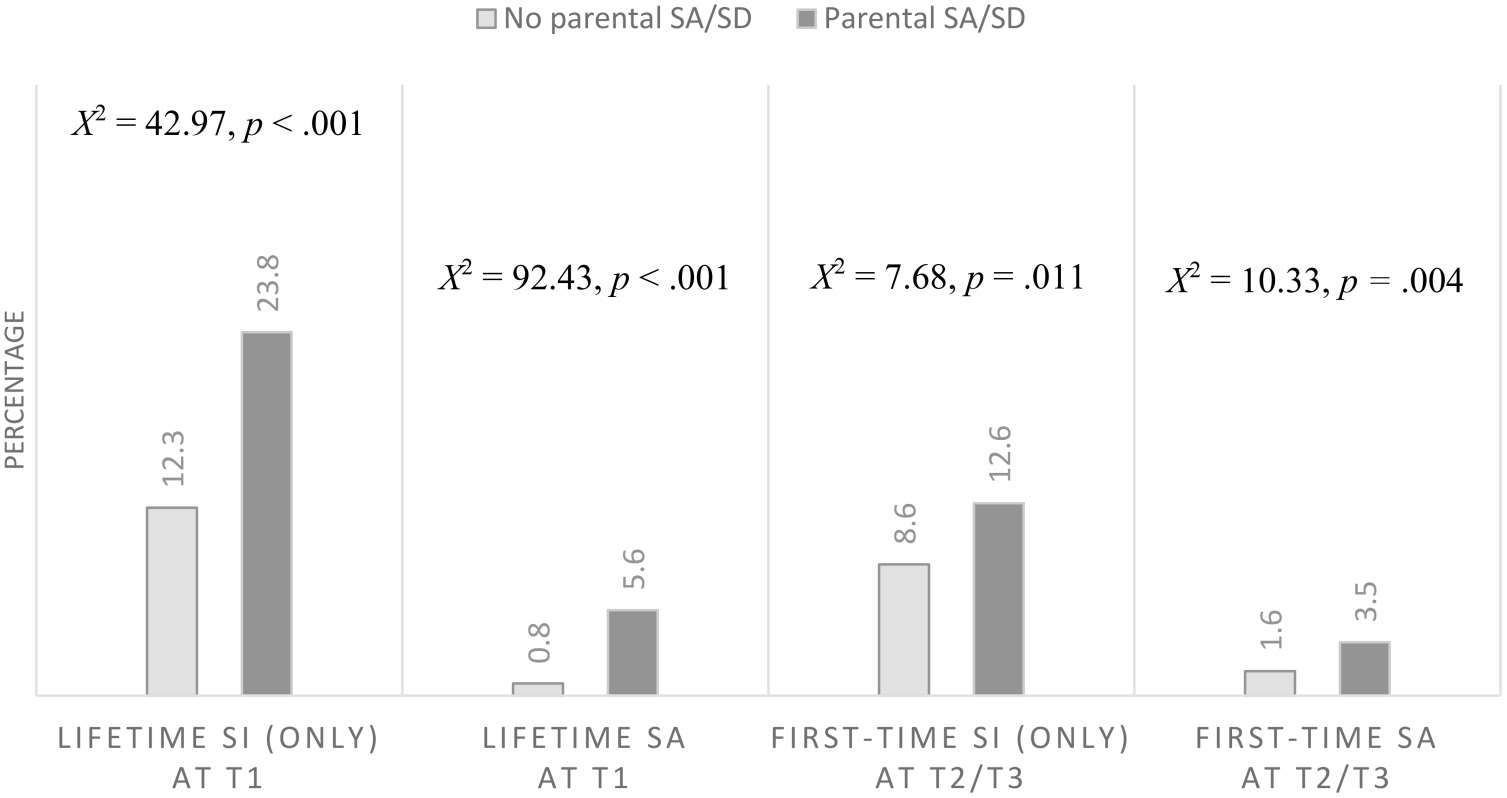
Association between parental suicide attempts or suicide death (SA/SD) and offspring suicide outcomes, suicide ideation (SI) and suicide attempt (SA), at each time point, Time 1 (T1) and Time 2 and/or 3 (T2/T3) (X axis). Second-order Rao-Scott adjusted Chi-square statistic reported. Percentages and crosstabs adjusted for weights and clusters. Light grey bars represent offspring whose parents did not attempt or die by suicide. Dark grey bars represent offspring whose parents attempted or died by suicide.

As **Figure 2** shows, a higher proportion of children with parental SA/SD reported lifetime SI and lifetime SA at T1, and first-time SI and first-time SI SA at T2/T3 compared to children whose parents have not attempted suicide or died by suicide. In models adjusted for the covariates, parental SA/SD remained associated with lifetime SI and lifetime SA at T1, and first-time SI at T2/T3 (**Table 2**).

**Table 2:** Association between parental SA/SD and offspring suicide outcomes.

	Time 1		Time 2 and/or Time 3	
	Lifetime SI (only)	Lifetime SA	First-time SI (only)	First-time SA
	OR (95% CI)	OR (95% CI)	OR (95% CI)	OR (95% CI)
Parental SA/SD	**1.64 (1.23, 2.19)**	**4.14 (2.57, 6.66)**	**1.45 (1.07, 1.95)**	1.47 (0.82, 2.64)

Significant results (p <.05) are bolded. SI, suicide ideation; SA, suicide attempt; SD, suicide death. Models adjusted for child’s sex, race/ethnicity, internalizing problems, family structure, financial adversity, weights, and clusters.

### 3.2 Confirmatory factor analysis

The five-factor structure of the Short UPPS-P Scale was identified using CFA. Based on the goodness-of-fit indices (**Supplementary Table 1**), three factors: lack of premeditation, positive urgency, and sensation seeking, needed to be modified. Following the procedure explained earlier (**Supplementary Tables 2, 3**), item 35 for positive urgency, item 16 for lack of premeditation, and item 21 for sensation seeking were removed^1^. The final loadings for each factor exceeded .40 (**Table 3**). The covariance matrix supported the discriminant validity of the five factors (**Supplementary Table 4**). The fit indices for the five-factor model were adequate (RMSEA= .030; CFI = .946; SRMR = .033).

**Table 3:** Factor analysis of the Short UPPS-P Impulsive Behavior Scale.

		Factor 1	Factor 2	Factor 3	Factor 4	Factor 5
Item 7	When I feel bad, I often do things I later regret in order to make myself feel better now.	.515				
Item 11	Sometimes when I feel bad, I keep doing something even though it is making me feel worse.	.525				
Item 17	When I am upset, I often act without thinking.	.508				
Item 20	When I feel rejected, I often say things that I later regret.	.657				
Item 35	When I am in a great mood, I tend to do things that can cause me problems.		–			
Item 36	I tend to act without thinking when I am very, very happy.		.686			
Item 37	When I get really happy about something, I tend to do things that can lead to trouble.		.715			
Item 39	I tend to lose control when I am in a great mood.		.713			
Item 6	I like to stop and think about things before I do it.			.665		
Item 16	I try to take a careful approach to things.			–		
Item 23	I am very careful.			.488		
Item 28	I tend to stop and think before doing things.			.834		
Item 15	I finish what I start.				.499	
Item 19	I tend to get things done on time.				.659	
Item 22	I am a person who always gets the job done.				.727	
Item 24	I almost always finish projects that I start.				.546	
Item 12	I enjoy taking risks.					.541
Item 18	I like new, thrilling things, even if they are a little scary.					.525
Item 21	I would like to learn to fly an airplane.					–
Item 27	I would like to ski very fast down a high mountain slope.					.435

Factor 1 = Negative urgency, Factor 2 = Positive urgency; Factor 3 = (Lack of) premeditation (reversed coded) Factor 4: (Lack of) perseverance (reversed coded); Factor 5: Sensation seeking.

### 3.3 Association between UPPS-P facets and SI (only) and SA

In unadjusted models (**Table 4**, Model 1), all UPPS-P facets (latent variables) were associated with lifetime SI and lifetime SA at T1. All facets, but sensation seeking, predicted first-time SI and first-time SA at T2/T3. These associations remained significant after adjusting for child’s sex, race/ethnicity, internalizing problems, family structure, and financial adversity (**Table 4**, Model 2). The associations between negative urgency and SAs at baseline and follow-up had the highest ORs and no overlapping confidence intervals with other UPPS-P facets (e.g., positive urgency, lack of premeditation).

**Table 4:** Association between UPPS-P facets (latent variables) and suicide outcomes in the offspring.

	Lifetime SI (only) at T1			Lifetime SA at T1	
	Model 1^a^	Model 2^b^	Model 3^c^	Model 1^a^	Model 2^b^
	OR (95% CI)	OR (95% CI)	OR (95% CI)	OR (95% CI)	OR (95% CI)
Negative urgency	**2.92 (2.29, 3.72)**	**2.60 (2.07, 3.27)**	**2.41 (1.62, 3.61)**	**10.66 (5.62, 20.22)**	**7.88 (3.95, 15.71)**
Positive urgency	**1.56 (1.37, 1.76)**	**1.49 (1.32, 1.69)**	0.90 (0.73, 1.13)	**1.87 (1.37, 2.55)**	**1.53 (1.16, 2.01)**
Lack of premeditation	**2.51 (2.12, 2.97)**	**2.23 (1.93, 2.59)**	**1.57 (1.32, 1.87)**	**2.78 (1.70, 4.55)**	**2.11 (1.35, 3.30)**
Lack of perseverance	**2.14 (1.73, 2.64)**	**1.80 (1.43, 2.25)**	1.21 (0.84, 1.73)	**4.30 (2.21, 8.35)**	**2.91 (1.60, 5.31)**
Sensation seeking	**1.48 (1.21, 1.82)**	**1.51 (1.23, 1.86)**	1.20 (0.92, 1.57)	**2.47 (1.30, 4.69)**	**2.82 (1.40, 5.68)**
	First-time SI (only) at T2/3			First-time SA at T2/3	
	Model 1^a^	Model 2^b^	Model 3^c^	Model 1^a^	Model 2^b^
	OR (95% CI)	OR (95% CI)	OR (95% CI)	OR (95% CI)	OR (95% CI)
Negative urgency	**1.81 (1.46, 2.25)**	**1.79 (1.46, 2.21)**	**1.70 (1.23, 2.35)**	**6.80 (4.52, 10.24)**	**6.44 (3.68, 11.28)**
Positive urgency	**1.33 (1.21, 1.46)**	**1.31 (1.20, 1.44)**	1.03 (0.85, 1.25)	**1.81 (1.26, 2.62)**	**1.60 (1.09, 2.37)**
Lack of premeditation	**1.56 (1.27, 1.92)**	**1.59 (1.30, 1.93)**	1.24 (0.92, 1.68)	**2.07 (1.43, 2.99)**	**1.86 (1.30, 2.65)**
Lack of perseverance	**1.64 (1.38, 1.94)**	**1.65 (1.39, 1.96)**	1.25 (0.93, 1.65)	**2.76 (1.81, 4.23)**	**2.18 (1.44, 3.29)**
Sensation seeking	0.96 (0.82, 1.13)	1.01 (0.85, 1.18)	0.87 (0.72, 1.05)	0.93 (0.54, 1.57)	1.11 (0.68, 1.82)

Significant results are bolded. SI, suicide ideation; SA, suicide attempt; T, time.

a Model 1: Adjusted for weights and clusters.

b >Model 2: Model 1 adjusted for child’s sex, race/ethnicity, internalizing problems, family structure, and financial adversity.

c Model 3: Model 2 adjusted for all the UPPS-P facets simultaneously.

When the five latent variables were entered simultaneously in the model (Model 3), negative urgency and lack of premeditation were independently associated to lifetime SI at T1. Negative urgency was the only facet that was uniquely associated with first-time SI at T2/T3 (**Table 4**, Model 3). Model 3 could not be run for lifetime SA at T1 and first-time SA at T2/T3 given the large standard errors, which is generally a result of a small number of cases where the dependent variable equals 0 or equals 1.

For informational purposes, the associations between negative urgency, all the covariates and each suicide outcome (**Table 4**, Model 2) are presented in **Supplementary Table 5**.

### 3.4 Mediation models

**Figure 3:**
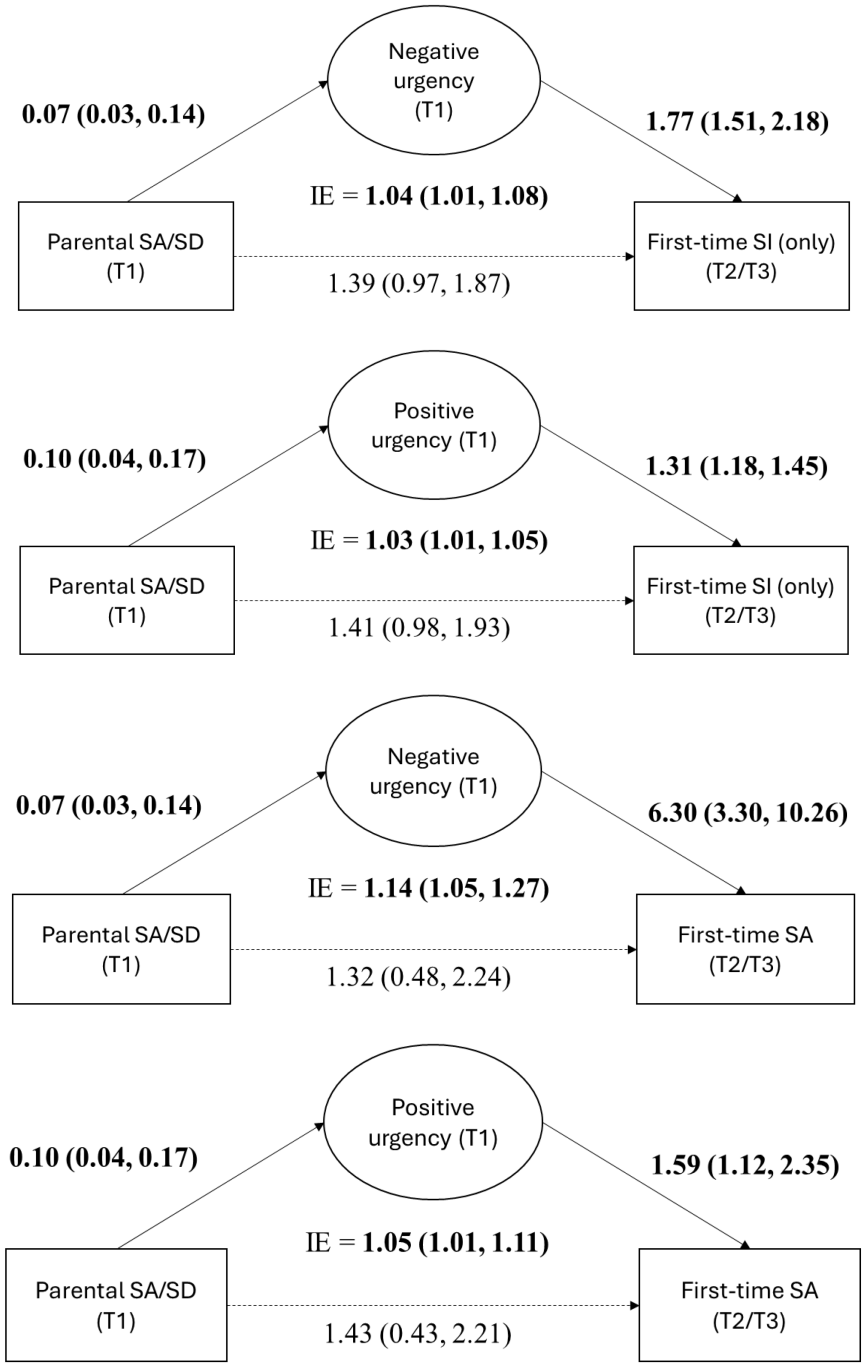
Mediation models showing the significant indirect effect (IE) of parental suicide attempts/suicide death (SA/SD) on first-time suicide ideation (SI) and suicide attempts (SA) thought negative and positive urgency on of the UPPS-P facets. The a path is reported as β (95% CI), and the b and c’ paths and the indirect effects are reported as OR (95% CI). Significant results are bolded. T, time.

Finally, we tested how the UPPS-P facets mediated the association between parental SA/SD and first-time SI (only) and first-time SA at T2/T3 (**Figure 1**; **Table 5**). In models adjusting for the covariates, parental SA/SD was associated with negative urgency and positive urgency (a path). Both negative and positive urgency were associated with first-time SI and first-time SA at T2/T3, when the models were further adjusted for parental SA/SD (b path). Parental SA/SD had a significant indirect effect on first-time SI at T2/T3 through negative urgency (OR = 1.04; 95% CI, 1.01-1.08) and positive urgency (OR = 1.03, 95% CI, 1.01-1.05). Parental SA/SD also had a significant indirect effect on first-time SA at T2/T3 through negative urgency (OR = 1.14, 95% CI, 1.05-1.27) and positive urgency (OR = 1.05, 95% CI, 1.01-1.11) (**Table 5**; **Figure 3**).

**Table 5:** Mediation models testing the indirect effect of the UPPS-P facets.

	Negative urgency	Positive urgency	Lack of premeditation	Lack of perseverance	Sensation seeking
Parental SA/SD → UPPS-P facet, β (95% CI)	**0.07 (0.03, 0.14)**	**0.10 (0.04, 0.17)**	0.04 (-0.01, 0.10)	0.03 (-0.004, 0.07)	0.04 (-0.02, 0.11)
UPPS facet → First-time (only) SI |adjusted for parental SA/SD, OR (95% CI)	**1.77 (1.51, 2.18)**	**1.31 (1.18, 1.45)**	**1.58 (1.24, 1.91)**	**1.65 (1.29, 1.86)**	1.00 (0.86, 1.18)
Parental SA/SD → First-time SI (only) | adjusted for UPPS-P facet, OR (95% CI)	1.39 (0.97, 1.87)	1.41 (0.98, 1.93)	1.42 (0.97, 1.96)	1.42 (0.99, 1.95)	**1.45 (1.01, 1.01)**
Natural indirect effect (bootstrapping), OR (95% CI)	**1.04 (1.01, 1.08)**	**1.03 (1.01, 1.05)**	1.01 (0.99, 1.05)	1.01 (0.99, 1.04)	1.00 (0.99, 1.01)
Parental SA/SD → UPPS-P facet, β (95% CI)	**0.07 (0.03, 0.14)**	**0.10 (0.04, 0.17)**	0.04 (-0.01, 0.10)	0.03 (-0.004, 0.07)	0.04 (-0.02, 0.11)
UPPS facet → First-time SA, adjusted for parental SA/SD | OR (95% CI)	**6.30 (3.30, 10.26)**	**1.59 (1.12, 2.35)**	**1.83 (1.24, 2.59)**	**2.16 (1.20, 2.88)**	1.12 (0.76, 1.85)
Parental SA/SD → First-time SA | adjusted for UPPS-P facet, OR (95% CI)	1.32 (0.48, 2.24)	1.43 (0.43, 2.21)	1.42 (0.59, 2.41)	1.41 (0.44, 2.31)	1.47 (0.44, 2.29)
Natural indirect effect (bootstrapping), OR (95% CI)	**1.14 (1.05, 1.27)**	**1.05 (1.01, 1.11)**	1.02 (0.99, 1.06)	1.02 (0.99, 1.06)	1.00 (0.99, 1.07)

Significant results are bolded. SI, suicide ideation; SA, suicide attempt; SD, suicide death. Each path was adjusted for child’s sex, race/ethnicity, internalizing problems, family structure, financial adversity, weights, and clusters.

## 4 Discussion

In this study, we examined the association between the UPPS-P facets and suicidal thoughts and behaviors and first-time endorsement within the next two years in a large US representative sample of children ages 9 to 10 years at baseline. In models adjusting for socio-demographic and clinical variables, all UPPS-P facets were cross-sectionally associated with SI and SA at baseline. At follow-up, all but sensation seeking, predicted first-time SI and first-time SA. However, when all facets were included in the same model, only negative urgency and lack of premeditation remained associated with lifetime SI at baseline and negative urgency with first-time SI at follow-up. In the mediation models, parental SA/SD had an indirect effect on first-time SI and first-time SA at follow-up through negative and positive urgency.

Our findings provide a close examination of the five impulsivity facets and contribute to clarifying their association with SI (only) and SA, cross-sectionally and prospectively, strengthening the conclusions that can be drawn about these associations. All UPPS-P facets were associated with SI and SA at baseline and follow-up, except for sensation seeking, after accounting for internalizing problems and other socio-demographic variables. As hypothesized, negative urgency emerged as an important UPPS-P facet, showing an independent association with SI (only), lifetime and first-time, and relatively strong associations with lifetime and first-time SA. Negative urgency captures the tendency to act impulsively when experiencing extreme negative emotions (33). Noteworthy, its association with SI and SA was minimally affected by the addition of internalizing problems to the model, which captured the presence of child’s anxiety, depressive and withdrawal symptoms, and somatic complaints in the past 6 months. Although internalizing problems and negative urgency are related (32, 80), it could be that children act impulsively when experiencing surges in negative affect (i.e., state) regardless of their daily levels of internalizing problems (i.e., trait). Our findings highlight the independent contribution of negative (and positive) urgency to suicide risk and point toward the need of providing children with specific strategies to utilize when they are experiencing surges in affect, regardless of their valence. Studies able to capture state *vs*. trait urgency or affect (e.g., ecological momentary assessment *vs*. one-time questionnaires) (81) may be able to further shed light on the association between negative urgency and suicide risk in childhood.

Additionally, poor emotion regulation strategies, problem-solving difficulties, and low distress tolerance have also been observed in individuals with elevated levels of negative urgency. These processes have been distinctively associated with the UPPS-P facets in mostly adult studies (41, 82–88). In a study with youth, ages 13-19, negative urgency was associated with less use of appropriate emotion regulation strategies and an increased use of inappropriate strategies, whereas lack of premeditation and perseverance were associated with an increased use of inappropriate strategies only (87). Among children with high negative urgency, it is possible that the desire to terminate their extreme negative emotions, in the context of experiencing emotion regulation or problem-solving difficulties to regulate them, may lead children to consider suicide or attempt suicide as a way to cope with the distress. Whether deficits in emotion regulation lead to higher levels of negative urgency (82) or vice versa (85, 86) has yet to be examined in longitudinal studies. It is possible that the direction of these associations varies by developmental period. The development of emotion regulation strategies and problem-solving skills begins in childhood and continues through young adulthood (89–91). Given impulsivity is a highly heritable trait that is especially elevated in childhood (19, 43), we could hypothesize that high levels of negative urgency may hinder the development of emotion regulation strategies and problem-solving skills, with subsequent bidirectional effects.

Lack of premeditation was also uniquely associated with SI (only) at baseline. Elevated levels of this facet may signal poor cognitive skills, such as difficulties disregarding non-relevant information (92, 93), which may prevent individuals from thinking about effective ways of solving their problems. Indeed, Valderrama et al. (2016) found that lack of premeditation, but not negative urgency, mediated the association between brooding (i.e., “dwelling on the reasons for one’s negative mood”) and suicide-related risk (47, p. 35). Although our findings regarding the independent association between lack of persistent and lifetime SI were cross-sectional, it could be that children with elevated lack of premeditation struggle to solve problems effectively. Difficulties in considering the potential consequences of their own actions to regulate their current behavior could lead to more maladaptive or risky behaviors and thoughts about suicide (32, 33). Future studies should examine how the UPPS-P facets distinctively relate to emotion regulation strategies and problem-solving skills and the direction of those associations in youth. Furthermore, how the UPPS-P facets interact with each other to increase suicide risk remains vastly unexplored.

Studies with clinical samples support the presence of high levels of impulsivity within families at risk for suicide (8, 11, 94). Our study was able to parse the UPPS-P facets associated with having a parent who has attempted or died by suicide, thus, those potentially involved in the familial transmission of suicide risk. Parental SA/SD was associated with negative and positive urgency in models that accounted for the covariates. In the mediation models, parental SA/SD had a significant indirect effect on first-time SI (only) and first-time SA at follow-up through both negative and positive urgency facets, although the effect size was small. It could be that parents who have attempted suicide or died by suicide are more likely to act impulsively when experiencing intense emotions. This emotion-based impulsivity may be transmitted from parents to offspring through genetic as well as environmental pathways and increase their suicide risk. In the emotion socialization process, children learn how to regulate their emotions through emotion-centered conversations with adults and observing how adults manage their emotions (i.e., modeling) and respond to the child’s own negative emotions (95, 96). If parents themselves have elevated levels of emotion-based impulsivity and associated deficits in emotion regulation strategies and problem-solving skills, this could impact how their children are learning to self-regulate within the home. Furthermore, parental non-supportive responses to adolescent’s negative emotions (e.g., punitive response) have been associated with SI in offspring (97). Given that parental SA and parental SD were asked with the same question, we could not disentangle their independent effects on the UPPS-P facets and suicide outcomes. Recent evidence suggests that these two experiences, and their timing of occurrence, may differently impact offspring mental health problems and suicide risk (28, 98, 99), and as such they could also have different effects on the UPPS-P facets. Futures studies should address this limitation and examine distinct pathways through which these experiences may increase suicide risk in the offspring and include different manifestations of externalizing problems, such as impulsivity, aggression, and inattention (22). Nonetheless, our findings suggest that urgency may be an early marker of risk for suicidal thoughts and behaviors in late childhood, especially among those children with parental history of SA/SD.

Finally, in line with prior studies (4, 5), parental SA/SD was associated with lifetime SI and SA at baseline and first-time SI during the two subsequent years. However, its association with first-time SA was further explained by socio-demographic and clinical factors. Future studies should test whether the prospective association between parental SA/SD and first-time SA emerges in adolescence, as SAs become more prevalent (100), or when an extended follow-up period is considered. Another point of exploration would be to test whether the identified associations between parental SA/SD, the UPPS-P facets, and offspring suicide risk change based on the severity of the SI, as the most frequent SI experienced by the children in this sample was passive SI (62), or the characteristics of the SA, such as the level of lethality or planning, which were not assessed in the ABCD study. Finally, future studies with this and other samples should ascertain whether the identified associations remain relevant considering the increase in mental health problems during adolescence.

This study has several limitations. As previously noted, one of the main limitations concerns the inability to disentangle the effects of parental SA from parental SD, as they were both asked in the same question. Recent evidence suggests that these two experiences may have a different impact on offspring mental health problems, making this an important question for future research. The attrition of mostly children of color or those with socio-economic problems may affect the generalizability of our findings to the general populations. Caregivers were not asked about child’s SI and SA during the 1-year assessment (T2), as such, a small proportion of children who did not report suicidal thoughts or behaviors during T2, but whose parents would have reported SI or SA, might have been misclassified. Our outcome was coded as first-time endorsement of SI and SA. While it does not necessarily correspond with the onset of those behaviors, it has clinical value as it captures the first time that children or caregivers were able or willing to disclose. Given the small percentage of children with SAs at baseline and follow-up, we could not run the models with all the UPPS-P facets for these outcomes. Finally, although most informants were biological parents (97.2%), it could be that some of the other informants were not aware about the history of SA/SD in the biological parents, leading to a slight underestimation of parental SA/SD.

## 5 Conclusions and clinical implications

In sum, our study examined the role of different facets of impulsivity on suicidal thoughts and behaviors in childhood and identified negative and positive urgency as facets that might underly the familial transmission of suicide risk. Our findings have direct implications for the identification, assessment, and intervention of children with suicidal thoughts and behaviors, especially among those with familial risk for suicide. Four out the five UPPS-P faces were associated with SI and SA. As such, across all children, prevention and intervention programs that target impulsivity and emotion regulation strategies, such as Parent–Child Interaction Therapy (101), have the potential to prevent or reduce suicide risk. One such program for schools is the Good Behavior Game, which has already shown promising effects in preventing suicidal thoughts and behaviors (102, 103).

For children whose parents have attempted suicide or died by suicide, elevated levels of emotion-based impulsivity may signal current and future suicide risk. Interventions indicated for these families could target emotion regulation strategies, problem-solving skills, and distress tolerance for both the caregiver and the children. Some UPPS-P facets seem to be more susceptible to change than others. Specifically, negative urgency and lack of premeditation have shown significant reductions after interventions (104, 105). These studies, although conducted with adults with substance use problems, show promising results for targeting the UPPS-P facets in interventions to prevent or reduce suicide risk in youth.

## Data Availability

Publicly available datasets were analyzed in this study. This data can be found here: NIMH Data Archive (NDA) (2021). Adolescent Brain Cognition Development (ABCD) Study. Curated Annual Release 4.0. https://dx.doi.org/10.15154/1523041. NIMH Data Archive (NDA) (2023). Adolescent Brain Cognition Development (ABCD)Study. Curated Annual Release 5.0. http://dx.doi.org/10.15154/8873-zj65.

## Footnotes

1 Watts et al. (2020) (63) tested the psychometric properties of the Short UPPS-P scale with this sample at T1. In their CFA, the authors also had to remove the item “I would like to learn to fly an airplane” given its low performance, which questions the validity of this item to assess sensation seeking in childhood. We removed two additional items, probably due to using a stricter RMSEA and differences in the sample size between the studies.
