# Supplementary material for: Negative and positive urgency as pathways in the intergenerational transmission of suicide risk in childhood
Source: Front Psychiatry. 2024 Sep 23;15:1417991. doi: 10.3389/fpsyt.2024.1417991 (PMC11456838; doi:10.3389/fpsyt.2024.1417991)
Supplement: Supplementary file 1 [file DataSheet1.pdf]

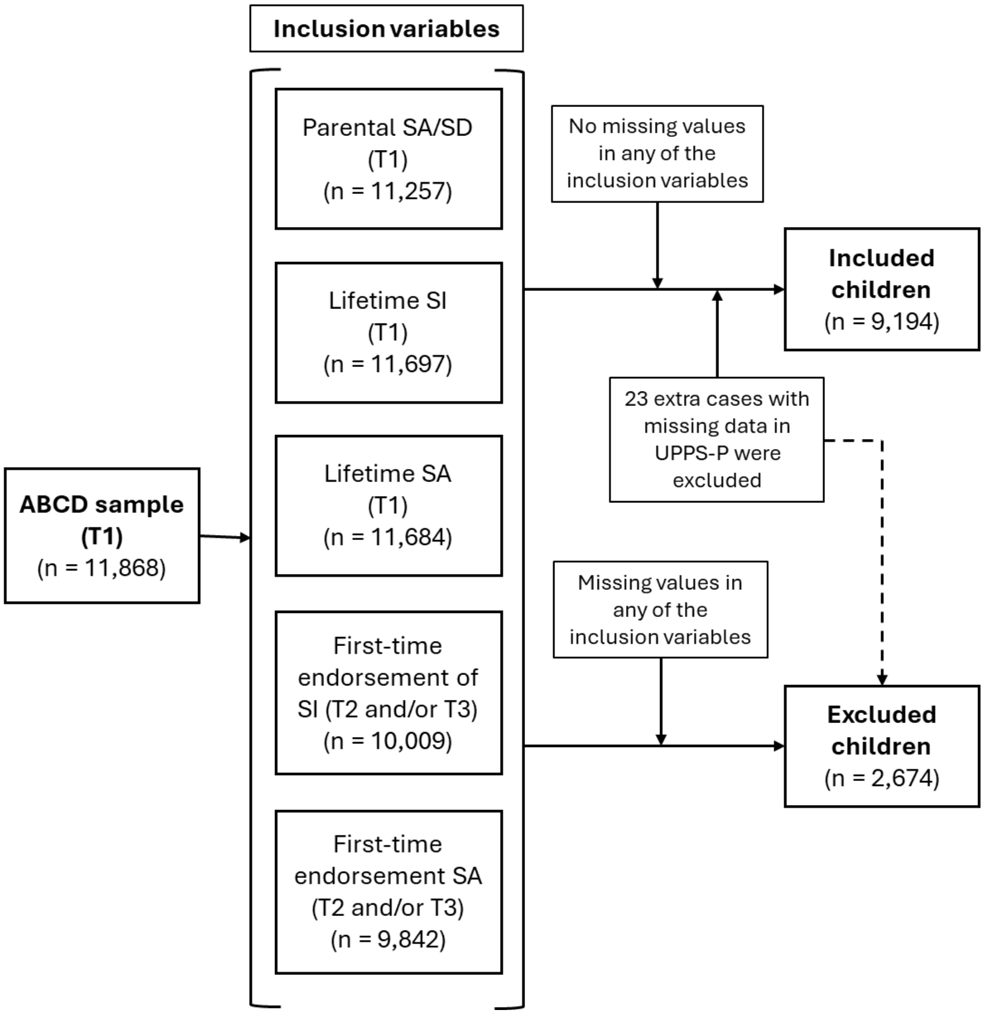

Figure S1. *Flow chart of participant inclusion and exclusion criteria. The sample size from the 4.0. data release was 11,877 children. Nine cases were removed from the dataset after the families withdrew consent to share their data. SI = suicide ideation; SA = suicide attempt; SD = suicide death; T = time.*

Table S1. Association between socio-demographic factors and offspring suicide outcomes.

	Time 1				Time 2 and/or T3			
	Lifetime SI (only)		First-time SA		First-time SI (only)		First-time SA	
	X^2	*p*	X^2	*p*	X^2	*p*	X^2	*p*
Sex	**16.96**	**< .001**	0.18	.672	**7.58**	**.012**	1.80	.194
Race/ethnicity	2.95	.052	1.55	.223	**2.96**	**.041**	1.98	.136
Family structure	**9.52**	**.006**	**13.68**	**.001**	0.56	.464	**20.04**	**< .001**
Internalizing problems	**436.22**	**< .001**	**172.13**	**< .001**	1.74	.202	**50.16**	**< .001**
COVID-19	1.32	.263	.003	.959	0.97	.336	1.27	.273
	t	*p*	t	*p*	t	*p*	t	*p*
Financial adversity	**-5.24**	**< .001**	**-4.19**	**< .001**	**-2.35**	**.028**	**-4.58**	**< .001**

Significant results ($p < .05$) are bolded. Second-order Rao-Scott adjusted Chi-square and t test statistics reported. Adjusted for weights and clusters. SI = suicide ideation; SA = suicide attempt.

Table S2. Goodness-of-fit indices for the UPPS-P factors.

	RMSEA <= .05	CFI >= .95	SRMR < .05
Negative urgency	.048	.984	.016
Positive urgency	.068	.978	.019
Lack of premeditation	.133	.859	.036
Lack of perseverance	.058	.958	.019
Sensation seeking	.114	.996	.045

Table S3. Standardized residuals for covariances.

Positive urgency	Item 35	Item 36	Item 37	Item 39	Loadings
**Item 35**	0.00				.633
**Item 36**	-0.26	0.00			.673
**Item 37**	30.68	0.00	0.00		.765
**Item 39**	-6.34	0.00	-0.17	0.18	.668
**Lack of premeditation**	**Item 6**	**Item 16**	**Item 23**	**Item 28**	**Loadings**
**Item 6**	0.00				.681
**Item 16**	-2.90	0.00			.524
**Item 23**	0.00	0.00	-0.59		.542
**Item 28**	0.00	-13.74	-4.09	0.072	.776
**Sensation seeking**	**Item 12**	**Item 18**	**Item 21**	**Item 27**	**Loadings**
**Item 12**	0.00				.426
**Item 18**	0.00	0.07			.425
**Item 21**	-7.80	-5.98	0.00		.337
**Item 27**	-3.16	-4.25	0.00	0.00	.609

Table S4. Correlation between the UPPS-P factors.

	1	2	3	4	5
1. Negative urgency	1	0.678	0.267	0.207	0.312
2. Positive urgency		1	0.265	0.232	0.327
3. Lack of premeditation			1	0.578	0.189
4. Lack of perseverance				1	-0.087
5. Sensation seeking					1

Table S5. Association between the covariates and each suicide outcome from Model 2 (Table 4) for negative urgency.

	Suicide ideation (only) T1	Suicide attempts T1	First-time suicide ideation (only) T2/T3	First-time suicide attempts T2/T3
	OR (95% CI)	OR (95% CI)	OR (95% CI)	OR (95% CI)
Negative urgency	**2.60 (2.07, 3.27)**	**7.88 (3.95, 15.71)**	**1.79 (1.46, 2.21)**	**6.44 (3.68, 11.28)**
Biological Sex	**1.23 (1.04, 1.46)**	0.81 (0.51, 1.29)	**0.77 (0.65, 0.90)**	**0.63 (0.44, 0.90)**
Internalizing problems	**3.70 (3.11, 4.42)**	**5.72 (3.83, 8.55)**	1.10 (0.90, 1.36)	**3.42 (2.15, 5.43)**
Financial adversity	**1.62 (1.08, 2.42)**	**3.11 (1.39, 6.96)**	**1.82 (1.12, 2.97)**	1.82 (0.83, 4.00)
Family structure	1.15 (0.96, 1.38)	**1.73 (1.05, 2.85)**	0.96 (0.82, 1.12)	**1.65 (1.10, 2.48)**
Non-Hispanic White	**1.40 (1.01, 1.93)**	0.74 (0.47, 1.16)	0.85 (0.67, 1.09)	0.96 (0.56, 1.64)
Hispanic	1.11 (0.85, 1.44)	0.88 (0.39, 1.95)	0.79 (0.62, 1.00)	1.27 (0.66, 2.43)
Non-Hispanic Other Races	**1.60 (1.19, 2.16)**	0.71 (0.26, 1.96)	**0.65 (0.44, 0.95)**	1.45 (0.71, 2.96)

Significant results are bolded. Covariates from Table 4 - Model 2 for negative urgency. Biological sex (‘female’ ref.); internalizing problems (‘no’ ref.); family structure (‘married/co-habiting’ ref.); race/ethnicity (‘non-Hispanic Black’ ref.).
